# Julia Bland interviews a group of anonymous (and frank) core trainees in psychiatry

**DOI:** 10.1192/pb.bp.115.052969

**Published:** 2016-08

**Authors:** Julia Bland

## How do core trainees see their jobs and the specialty?

Given the crisis in recruitment into psychiatry, I wanted to hear how a group of CT1 and CT2 psychiatrists perceived their chosen specialty. Could they recommend it?

Perhaps typically of a group of doctors, the first flavour was cynicism. The jokey reply to the question ‘Why choose psychiatry?’ is ‘To avoid med regging’, but as the conversation unfolded it became safe to admit ‘I really love my job’. The group was disparate in terms of age, experience, nationality, culture and, of course, the particular jobs they were doing. During the first 2 years of psychiatric training they see how four different teams operate, community or ward based, and then the on-call experience offers a rich mix of interest, alarm and frustration. A bright and curious bunch, they are rapidly accruing a database of experiences, exchanging impressions with each other, checking and observing themselves and their peers. In this early stage there is a looking back at the culture of medical wards and comparing medicine and psychiatry.

‘The dynamics between the doctors and the nurses are very different on psychiatric wards compared [with] medical ones … the nurses are more in charge … there is less hierarchy, it's more friendly, and the nurses know how to do some things much better than us, for example, how to restrain a patient safely … ’.

## What did they hope for in choosing psychiatry?

‘I wanted a reasonable amount of time with patients, less of a checklist, the opportunity to develop a relationship with patients … ’.

This hope was not always fulfilled:

‘It's been disappointing. I do mainly the physical [assessment], medical stuff and paperwork … ’.

‘I have no time to speak to patients. We've only got one SHO [senior house officer] and no reg[istrar], I only see them in ward rounds, we have to aim to discharge one patient every day’.

Others did manage the hoped-for patient contact:

‘My ward is great for exposure to patients. We do interviews in the ward rounds, and we are encouraged to prioritise time on the ward. It's rewarding when patients tell you something different, something that they haven't told anyone else. But then my ward is notorious for long admissions … ’.

Rapid turnover and broken continuity was a theme of criticism:

‘It's frustrating when patients are suddenly moved, even after you've put time in … and if the patients go on home leave, or overnight leave for more than 2 days, the bed goes. It's bad for the patients and for us, the lack of continuity’.

Another theme was medication as a speedy response with lack of time for psychological therapies:

‘The attitude of my consultant is “Let's give you [the patient] more antipsychotics to decrease risk and get you out”.’

Another states:

‘I was surprised, I thought there would be a bit more psychotherapy, there is no time for the ward psychologist to give my patient CBT’.

More alarmingly, one CT reported:

‘I was mocked for asking about a dynamic formulation … The theme is “We're scientists” but meds don't do very much a lot of the time. I'm worried that with the cuts we don't have the luxury of thinking, and the biological model is winning … ’.

## How would they like psychiatry to evolve?

‘I would like more prevention … but I can't see it happening’

‘There needs to be more continuity … a patient told me she had had the same CPN [community practice nurse] for 2 years, but now there's such a turnover’.

‘I want less time on the computer, I'd say it's 50 to 80% of my day. It's a poor balance, very documentation heavy, with a lot of arse covering. I know it's a bit of an SHO sickness, and some longer entries are valuable, but there is a lot of cut and pasting in the records … ’.

‘There should be less dependence on locums: they can do a week a month and earn as much as us.’

‘I'm annoyed by the use of private beds … but it's scary that really sick patients are out there waiting to be admitted’.

But this was not just moaning or disappointed idealism; there was enthusiasm as well:

‘The consultants do know what's going on with the patients, unlike on medical wards … ’

‘Psychiatrists have a broader view of the world, and psychiatric colleagues are easier to get on with, they are more supportive, less competitive and with much wider interests.’

‘The good nurses are amazing … they are compassionate with horrible, frightening patients’.

The trainees are not in a hurry to become consultants – ‘being a consultant in the community would be terrifying’ – and they agreed that ‘there is an argument for dragging out the training … we are lucky to have protected training time.’ One stated: ‘I am seriously considering an Associate Specialist route, so that I can have less admin and more direct clinical time.’

The course of the interview was in the shape of a bowl. We sank down to the bottom of criticism and disappointment to end on the upbeat statement: ‘Doing psychiatry is the best work-related decision I've ever made’.

From the perspective of someone much later in their career, they are an impressive group who are fired with passion for their patients' welfare, if struggling with the reality of coalface practice. I was left with optimism for a profession that is still attracting such thoughtful, funny and brave people to its ranks.

